# Fluorescence lifetime imaging microscopy approach reveals quantitative signatures for hepatocellular carcinoma diagnosis

**DOI:** 10.3389/fonc.2025.1598334

**Published:** 2025-07-29

**Authors:** Lingyun Li, Aoshan Wang, Xiongqing Liu, Ganlu Wang, Shanjie Xu, Changjiang Li, Wenzhong Wu, Xiaoying Zhang, Zhendan He, Huiling Qiu, Xiao Peng, Wei Yan, Junle Qu

**Affiliations:** ^1^ School of Pharmacy, Shenzhen University Medical School, Shenzhen University, Shenzhen, Guangdong, China; ^2^ College of Pharmacy, Shenzhen Technology University, Shenzhen, China; ^3^ College of Physics and Optoelectronic Engineering, Key Laboratory of Optoelectronic Devices and Systems of Ministry of Education and Guangdong Province, Shenzhen University, Shenzhen, China; ^4^ Hepatobiliary and Gastrointestinal Surgery, Fuyong People's Hospital of Baoan District, Shenzhen, China

**Keywords:** hepatocellular carcinoma (HCC), fluorescence lifetime imaging microscopy (FLIM), hematoxylin and eosin (H&E), diagnostic accuracy, microenvironment

## Abstract

Hepatocellular carcinoma (HCC) remains a critical global health challenge, and current histopathological diagnosis relies heavily on hematoxylin and eosin (H&E) staining—a widely adopted clinical tool for assessing tissue morphology. However, H&E staining alone cannot provide quantitative data for diagnosis of tumor samples. Poorly differentiated or unclear lesions are difficult to distinguish. Pathologists often need to rely on subjective judgment. Additional immunostaining is usually required to confirm the diagnosis. In this work, we have applied fluorescence lifetime imaging microscopy (FLIM) method into detecting H&E staining HCC tissue sections. This method provided the eosin fluorescence information of tissue sections, resulting in improved diagnostic accuracy and efficiency. We employed FLIM to compare the fluorescence lifetime distributions between the cancerous regions and the corresponding peritumoral regions. These results demonstrated that the fluorescence lifetime values in cancerous tissues significantly exceeded those of peritumoral region tissues, with their averages ranging from 2000–2500 picoseconds (ps) compared to 500–1000 ps in peritumoral region tissues. This finding has indicated higher fluorescence lifetime values of the fluorescent molecules in cancerous regions, suggesting distinct microenvironment of these regions. Furthermore, correlation analysis was applied between the ratio of fluorescence lifetime values and a series of liver function indicators, such as total bilirubin and transaminases, suggesting potential biochemical markers for clinical monitoring and diagnosis of HCC. The synergistic use of FLIM and H&E staining can bridge morphological and functional characterization, providing a quantitative method to investigate HCC microenvironments. This approach not only preserves the diagnostic utility of H&E but also adds metabolic profiling capabilities, facilitating deeper mechanistic exploration of tumor progression. Future work can be explored into integration and optimization of FLIM-H&E protocols in larger samples for further clinical diagnosis.

## 1 Introduction

Hepatocellular carcinoma (HCC) is a prevalent and aggressive form of cancer that poses a significant threat to human health. In 2018, HCC was the sixth most common cancer and the fourth leading cause of cancer-related deaths worldwide (1). HCC typically develops in patients with cirrhosis and is linked to a dismal prognosis, with a 5-year survival rate of only 18% (2).

Early and accurate diagnosis is essential for improving outcomes in HCC. Current diagnostic approaches—including serum biomarkers such as alpha-fetoprotein (AFP) (3), imaging techniques (4, 5), and histopathological examination (6, 7)—remain limited in terms of sensitivity, specificity, and diagnostic consistency. AFP levels can also be elevated in certain benign liver diseases, reducing its reliability in distinguishing malignant from non-malignant conditions (8, 9). Imaging modalities such as ultrasound and computed tomography (CT) are commonly used to detect tumor size and location but often fail to reliably differentiate between benign and malignant lesions (5, 10). Although magnetic resonance imaging (MRI) provides excellent soft tissue contrast, its high cost limits its routine use in HCC diagnosis (11).

To address these gaps, modern technologies such as contrast-enhanced ultrasound and artificial intelligence (AI)-assisted imaging are being explored (12). Nanoparticle-based contrast agents enhance imaging sensitivity and tumor targeting across modalities (13), while AI improves diagnostic accuracy and reduces operator dependence (14). For example, integrating nanomedicine (e.g., Rutin-loaded nanoparticles) enables image-guided drug delivery and real-time monitoring, enhancing both diagnosis and therapy (15). However, challenges in resolution, standardization, and tissue-level precision remain in this field.

Currently, histopathological examination remains the primary method for diagnosing HCC (16, 17). Hematoxylin and eosin (H&E) staining is a widely used method that provides information on tissue cell morphology and structure (16, 17). However, H&E staining alone may not provide an accurate diagnosis, particularly in poorly differentiated tumors or those resembling other liver diseases. In these situations, the accuracy of the differential diagnosis often relies on the pathologist’s expertise, which may result in variability in diagnosis. Consequently, additional staining or immunostaining is often necessary to accurately identify tissue components in H&E-stained biopsy samples. This process is constrained by the spectral properties of available fluorophores, the limited amount of fluorochromes that can be bound in a single stain, and the availability of commercially produced antibodies (18–20). Furthermore, H&E staining and immunofluorescence are usually conducted on separate tissue sections, which may result in the loss or distortion of critical morphological details in irregular areas, potentially less accuracy of diagnostic information (21, 22). Therefore, there is a need for a simplified diagnostic approach that can mine more precise information from H&E staining in a quantitative way.

Fluorescence lifetime imaging microscopy (FLIM) is an advanced imaging method that offers more detailed information than traditional histopathology (23). FLIM differs from conventional imaging techniques with the ability of extracting *in situ* fluorescence lifetime information, which may indicate changes in the surrounding environments, regardless of excitation power, fluorophore concentration, or photobleaching (24, 25). In this study, we have used time-domain FLIM technique to directly measure fluorescence lifetime values from H&E-stained HCC slides and applied the phasor approach into fluorescence lifetime analysis to enhance the visualization of pathological characteristics. The phasor approach to fluorescence lifetime can be used to study different types of tissues in H&E-stained HCC sections to generate immunostaining-like multicolor images, thus providing an intuitive method for histopathology examination *in situ*.

Here, we have applied FLIM into analyzing the fluorescence lifetime values of H&E-stained HCC slides. The phasor method was further performed for data analysis. This approach has enhanced the visualization of pathological features and enables the analysis of various tissue types in H&E-stained HCC sections, producing images com-parable to immunostaining. In summary, FLIM method can serve as a straightforward *in situ* diagnostic tool for histopathology.

## 2 Materials and methods

### 2.1 Sample preparation

Four fresh liver specimens were obtained from patients undergoing routine liver biopsies at the Department of Hepatobiliary and Gastrointestinal Surgery, Fuyong People's Hospital, Baoan District, Shenzhen. The specimens were placed in standard pathology transport containers, kept on ice, and promptly delivered to the Department of Pathology.

Each specimen was processed using standard histological procedures. Paraffin-embedded tissue blocks were first trimmed using a rotary microtome to expose the tissue area, leaving a 1–2 mm margin to preserve tissue integrity. The section thickness was set to 4 μm. Serial sections were cut by using a glass knife at a consistent speed to minimize wrinkles. The resulting paraffin ribbons were floated in a 40–45 °C warm water bath to fully expand the tissue. Expanded sections were then mounted onto microscope slides, ensuring the tissue was centered. After draining excess water, the slides were baked in a 60 °C oven for 30–60 minutes to ensure firm adhesion of the sections.

A senior pathologist performed histopathological evaluation of the H&E-stained liver sections, confirming the diagnosis of HCC. One H&E-stained section from each patient was selected for subsequent imaging analysis.

This study was conducted in accordance with a protocol approved by the Research Ethics Committee of Fuyong Peoples's Hospital of Baoan District, Shenzhen. Informed consent was obtained from all patients for the use of their tissue samples in medical research.

For clarity, the four specimens were labeled H1, H2, H3, and H4. According to the China Liver Cancer Staging system, H1 was classified as stage IIA, H2 and H3 as stage IB, and H4 as stage IA, respectively. Relevant pathological data for each specimen are presented in **Table 1** in the Results and Discussion section. The laboratory indicators listed in **Table 1** are all derived from preoperative test results, specifically from blood samples collected within 24 hours of patient admission. This time point was chosen to ensure that all clinical parameters accurately reflect the patient's physiological status prior to surgery, thereby providing a consistent and reliable basis for correlating pathological findings with FLIM data.

**Table 1:** Basic characteristics of HCC patients and correlation analysis.

Sample	CNLC	Lifetime Ratio (Cancerous / peritumoral)	Prothrombin Time (s)	Total Bilirubin (µmol/L)	Direct Bilirubin (µmol/L)	Indirect Bilirubin (µmol/L)	ALT (U/L)	AST (U/L)	Total Plasma Protein (g/L)	Albumin (g/L)	Globulin (g/L)	Tumor Marker AFP (ng/mL)
H1	IIA	1.79	13.6	69.7	16.3	53.4	28	38	70.7	39.2	31.5	1079
H2	IB	1.12	15	8.7	4.3	4.4	21	59	52	31.5	20.5	34072
H3	IB	1.38	12	7.1	0.9	6.2	47	43	74.1	43.9	30.2	25.3
H4	IA	1.77	15.7	30	8.6	21.4	23	36	57.3	28.1	29.2	463.1
Correlation Analysis			0.63	0.79	0.87	0.75	-0.68	-0.53	-0.23	-0.54	0.29	-0.10

H1, H2, H3, and H4, hepatocellular carcinoma (HCC) patients. CNLC, China Liver Cancer Staging; IA: Single tumor ≤ 5 cm, no vascular invasion, no metastasis, Child-Pugh A; IB: Single tumor > 5 cm, no vascular invasion, no metastasis, Child-Pugh B; IIA: 2–3 tumors, each ≤ 3 cm, no vascular invasion, no metastasis, Child-Pugh A;

ALT, Alanine aminotransferase; AST, Aspartate aminotransferase; AFP, Alpha-fetoprotein.

### 2.2 Experimental equipment

The section samples were observed by using the FLIM system (DCS-120, Becker & Hickl, GmbH, Germany), which featured a confocal microscope, the Eclipse TE2000-U (Nikon) with a supercontinuum white laser (SC-PRO-7, YSL Inc., China). The system used a Time-Correlated Single Photon Counting (TCSPC) mode (Becker & Hickl GmbH, Germany) with a highly sensitive hybrid detector (HPM-100, Becker & Hickl GmbH, Germany) (26).

The fluorescence lifetime at each pixel of a 256×256 image is calculated for all stained tissue sections using biexponential fitting, expressed as follows:


(1) $$\frac{I\left(t\right)}{I\left(0\right)}=\;a1\;exp\left(\;t/\tau 1\right)+\;a2\;exp\left(\;t/\tau 2\right)$$

In this Equation 1, τ1 and τ2 denote the lifetimes of the two components, while a1 and a2 represent their respective amplitudes. The average lifetime (τm) for each pixel is determined using the following Equation 2:


(2) τm= (a1τ1+a2τ2)/(a1+a2)

A pseudo-colored lifetime image is generated by assigning distinct colors to the τm values of each pixel. Lifetime fitting and calculations are performed by using SPCImage software (Becker & Hickl GmbH, Germany), which is designed for TCSPC-FLIM data analysis and lifetime fitting using the SPC-150 module. Bright-field images can be captured with a digital camera (Leica DFC310 FX CCD).

### 2.3 FLIM data acquisition

Images of each sample was obtained from the DCS120 system using a 40× objective, with an excitation wavelength of 540 nm and an emission filter of 620/60 nm according to our previous work (27, 28).

The excitation wavelength of 540 nm was selected because it closely matches the excitation peak of the main fluorescent components in the samples, such as the dyes used in H&E staining. This effectively excites the fluorescence signal and enhances the signal-to-noise ratio. The paired 620/60 nm emission filter efficiently blocks stray light and selectively captures the emission band of the target fluorescence, ensuring accurate and sensitive detection (27).

Each image was then analyzed with SPCImage software, generating FLIM data with distinct colors corresponding to different lifetime values. This analysis can result in a pseudo-colored image on the left as well as a lifetime distribution histogram in the upper-right region (**Supplementary Figure S3**). In the following analysis, the lifetime range displayed in the pseudo-colored lifetime image was set from 500 ps to 3500 ps. The color distribution was arranged from shortest to longest lifetime, in the following order: blue, green, and red.

### 2.4 Phasor approach for FLIM data analysis

To gain further insights, phasor plot analysis is performed using a Fourier trans-form. This method converts the time-domain data of each pixel into polar coordinates, with phase and amplitude values indicated as S for the vertical axis and G for the horizontal axis as shown in the following Equations 3 and 4.


(3) $$S_{i,j}\left(\omega \right)=\frac{\int_{0}^{\infty }I\left(t\right)\sin\left(n\omega t\right)dt}{\int_{0}^{\infty }I\left(t\right)dt}$$

(4) $$G_{i,j}\left(\omega \right)=\frac{\int_{0}^{\infty }I\left(t\right)\cos\left(n\omega t\right)dt}{\int_{0}^{\infty }I\left(t\right)dt}$$

In this context, i and j denote the pixel coordinates in the image. Si,j(ω) and Gi,j(ω) indicate the vertical (y) and horizontal (x) coordinates of the phasor plot, respectively. Furthermore, in this study, ω = 2πf, where f is the laser repetition frequency of 76 MHz. The harmonic order was denoted by n, with n=1. The phasor distribution is analyzed by using cluster identification according to our previous work (27).

There is a direct connection between phasor location and fluorescence lifetime. Each lifetime corresponds to a unique point on the phasor plot, which universally rep-resents decay. All single exponential lifetimes lied on the “universal circle”, which is a semicircle with a radius of 1/2, stretching from point (0,0) to point (1,0). In this context, the point (1,0) indicates τ=0, while (0,0) represents τ=∞.

In phasor space, two single-lifetime components can be combined directly through vector algebra. A mixture of two distinct single-lifetime components, both situated on the universal semicircle, will align along the chord that connects their points. Pixels that share identical fluorescence lifetime components cluster at the same location on the phasor plot.

Clustering assignments involved selecting distinct groups within the phasor plot. Each group represents pixels with similar fluorescence lifetime characteristics in the image. Adjustments to the separation of clusters considered the spatial distribution and morphological features of different cellular substructures or tissues.

### 2.5 Correlation analysis

Pearson correlation analysis (denoted as r) was employed in order to examine the relationship between variables. The value of the Pearson correlation coefficient, r, ranges from -1 to +1. A Pearson correlation coefficient (r) of +1 signifies a perfect positive linear correlation. This means that both variables increase together. An r value of -1 signifies a perfect negative correlation meaning that as one variable increases, the other decreases, while an r value of 0 indicates no linear correlation.

## 3 Results and discussion

**Figure 1:**
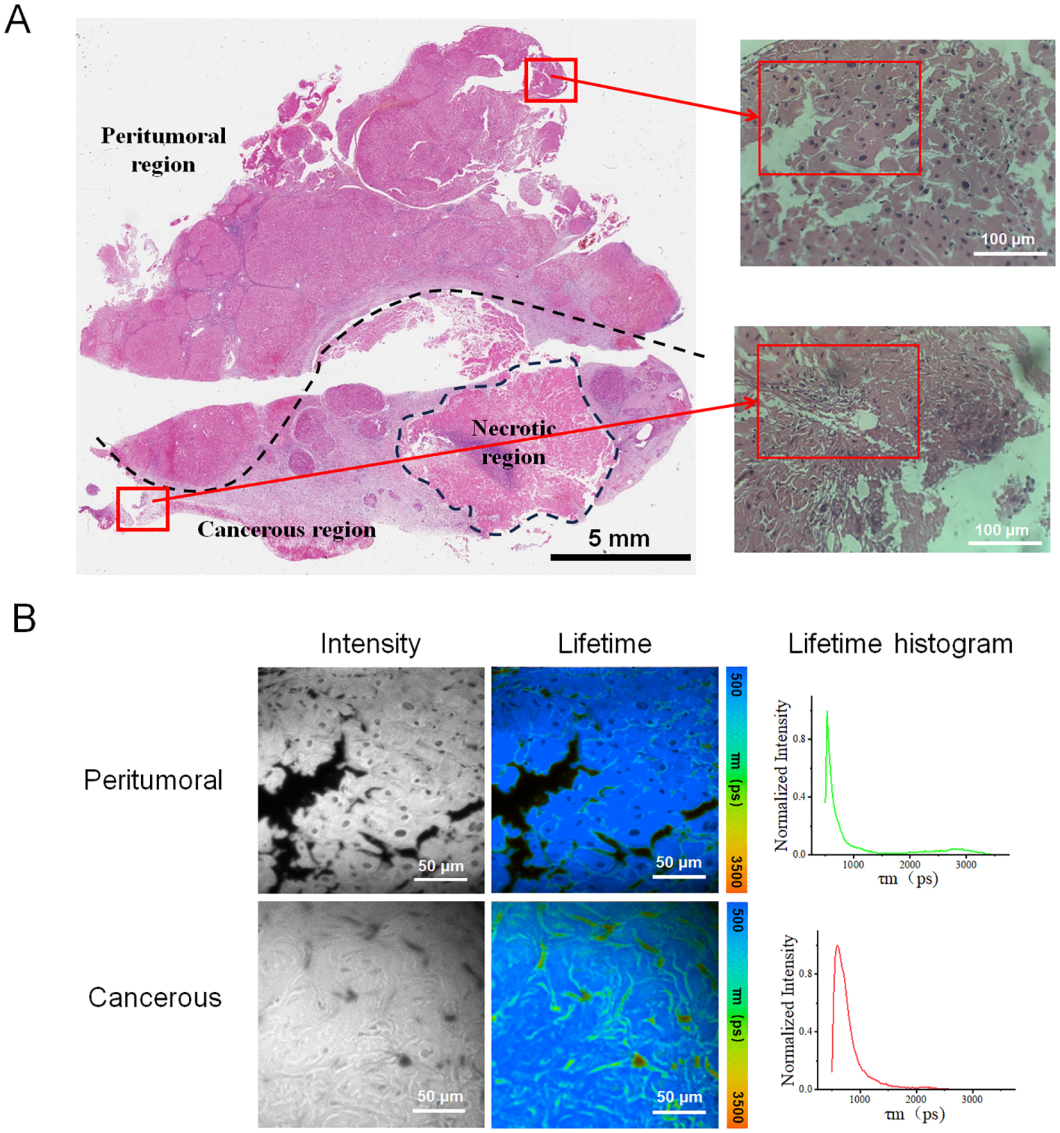
**(A)** Tissue section labeled H1 depicted the liver cancer region and the surrounding peritumoral region, including an annotated necrotic site. The left image showed the whole-slide scanned image, featuring a 5 mm scale bar. The two right images displayed the peritumoral and cancer regions, corresponding to the upper and lower red boxes in the left image. The inner red boxes highlight the regions selected for FLIM data collection. **(B)** Fluorescence lifetime images were obtained from the peritumoral and cancerous regions of sample H1. These images included the intensity images, fluorescence lifetime pseudo-colored images, and lifetime distribution histograms associated with the corresponding fluorescence lifetime images.

To determine whether the processed data can effectively distinguish the liver cancerous region from peritumoral region, the slice of sample H1 was imaged as an example, as shown in **Figure 1**. The bright-field image of the entire slice, delineating the cancerous and peritumoral region, was presented in **Figure 1A**. The representative regions from both cancerous and peritumoral region were selected to obtain FLIM data. The images of fluorescence intensity and lifetime of each region were presented in **Figure 1B**. The histograms indicated that the lifetime of the cancerous region has the property of a wider, multi-peaked distribution with longer average lifetime values. In contrast, the lifetime distribution of the surrounding peritumoral region showed a narrower distribution with shorter lifetime values. This difference might indicate the distinct molecular environments and metabolic activities between these two types of regions.

**Figure 2:**
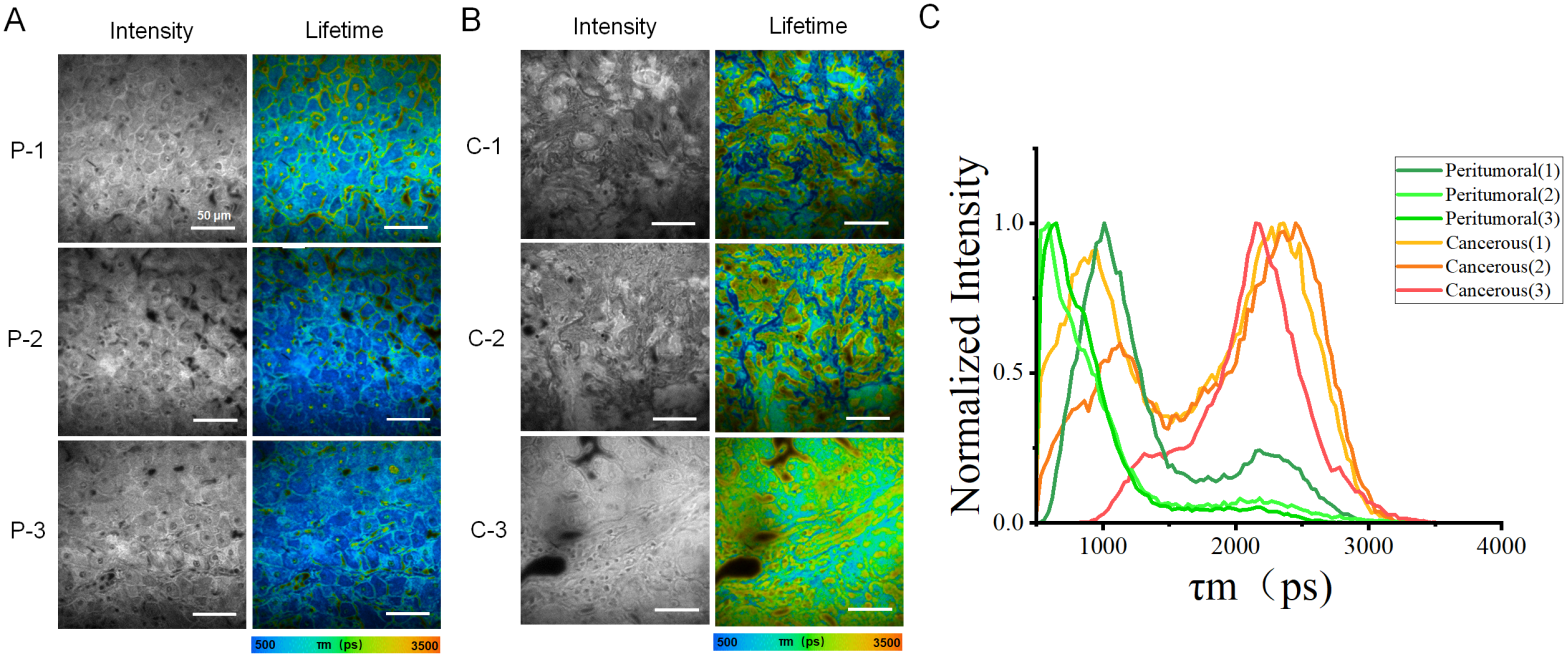
Images and distribution of fluorescence lifetime in peritumoral and cancerous tissues. **(A)** Rows (P-1, P-2, and P-3) showed the intensity images of peritumoral regions along with the corresponding fluorescence lifetime images. **(B)** Rows (C-1, C-2, and C-3) displayed microscopic images of cancerous tissue. These include intensity images of the peritumoral regions, as well as the corresponding fluorescence lifetime images. **(C)** The fluorescence lifetime distribution histograms of all peritumoral and cancerous tissues. The red, orange, and yellow curves represented cancerous tissue, while the green curves indicated peritumoral tissue. Scale bar=50 μm.

After analyzing the collected data, fluorescent images and their corresponding lifetime distribution curves were generated from three peritumoral regions and three cancerous regions of sample H1, as illustrated in **Figure 2**. **Figure 2C** showed that the fluorescence lifetime values in the cancerous regions were significantly higher than those in the peritumoral regions. The curve for the cancerous region exhibited a distinct peak in the longer lifetime range (2000–2500 ps), indicating a higher prevalence of molecules with longer fluorescence lifetimes in the cancerous tissue. By contrast, the curve for the peritumoral samples revealed a higher proportion of shorter lifetimes (500–1000 ps), suggesting that short-lifetime molecules were more abundant in this tissue.

**Figure 3:**
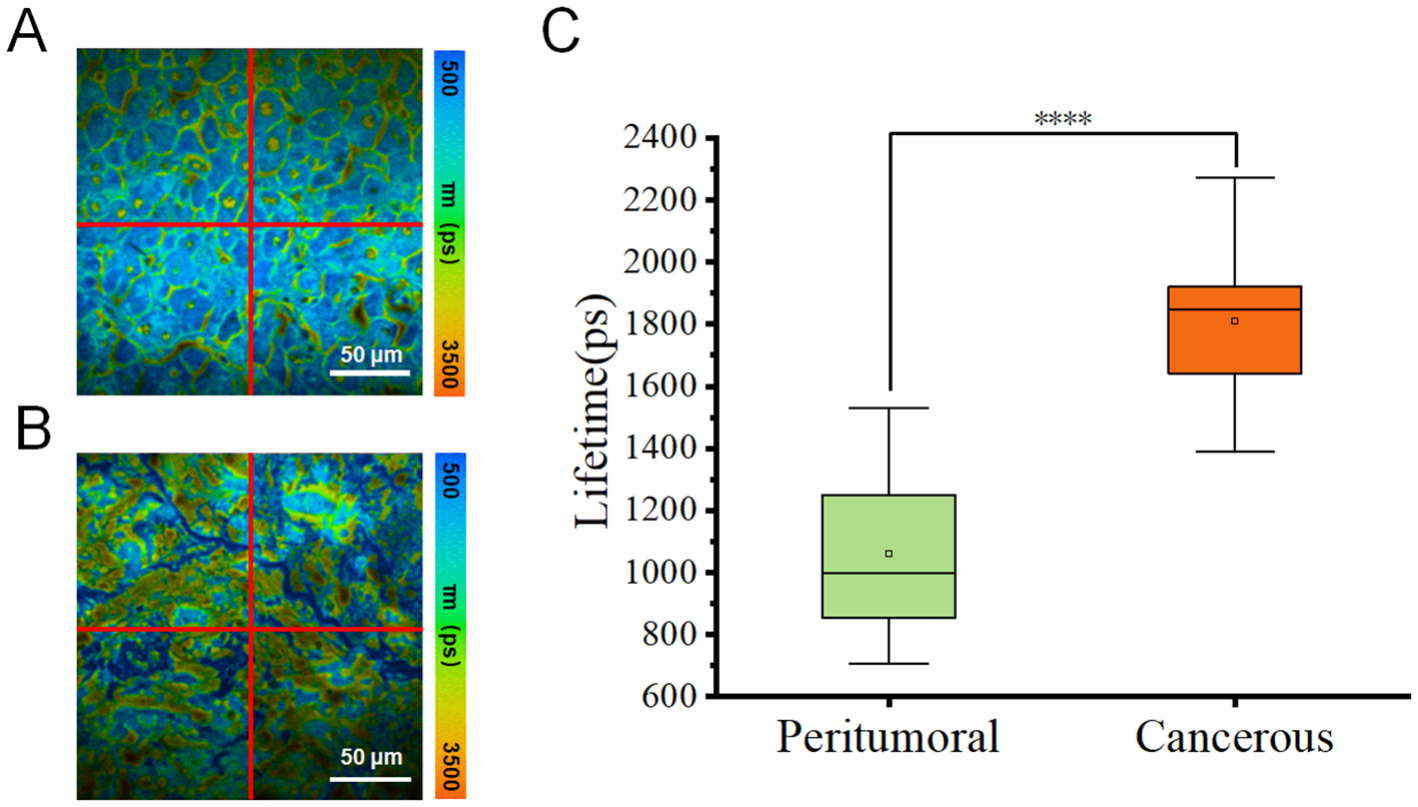
**(A, B)** Pseudo-color images of one representative fluorescence lifetime from either peritumoral region **(A)** or cancerous region **(B)** were segmented into four quadrants (1, 2, 3, 4) in a clockwise manner. Scale bar=50 μm. **(C)** Comparison of fluorescence lifetimes peritumoral and cancerous tissues. The combined box plot displayed the fluorescence lifetime distribution for peritumoral (n=12) and cancerous (n=12) regions. The data showed fluorescence lifetime values from all quadrants, highlighting a significant difference between the two groups (****p < 0.0001).

To improve the data analysis, each fluorescence lifetime pseudo-colored image was divided into four sections (**Figure 3A**), whose data were used to generate box plots (**Figure 3B**). **Figure 3C** showed that the fluorescence lifetime in cancerous regions ranged from approximately 1400–2300 ps, which was higher than in other areas. Both the median and mean values were significantly higher than those of the peritumoral tissues. In contrast, the fluorescence lifetime in the peritumoral tissue was lower, ranging of approximately 700–1500 ps, indicating a shorter overall fluorescence lifetime. Additional validation confirmed that cancerous tissues exhibited a higher average fluorescence lifetime. Both the median and mean values of cancerous tissues were significantly greater than those of the peritumoral tissues. The student t-test was conducted on 12 datasets obtained from both the cancerous and adjacent peritumoral regions. The data represented fluorescence lifetime values obtained from all quadrants, demonstrating a significant difference between the cancerous and peri-tumoral regions (****p < 0.0001).

**Figure 4:**
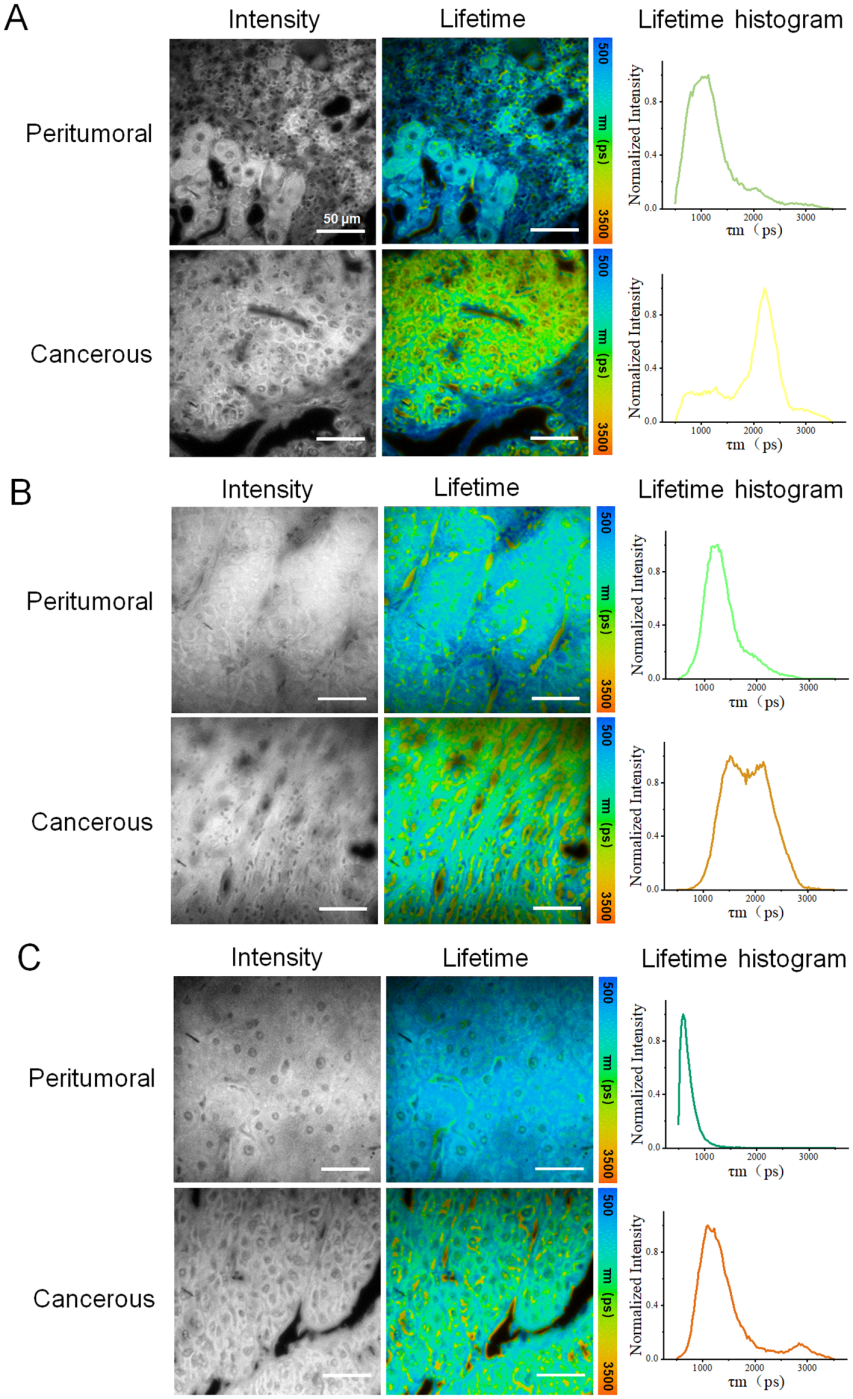
Fluorescence lifetime imaging (FLIM) analysis of three other HCC tissue samples: **(A–C)** correspond to samples H2, H,3 and H4, respectively, with data acquired from peritumoral and cancerous regions. Each row of data included an intensity image, a fluorescence lifetime pseudo-colored image, and a lifetime distribution histogram. For each sample, one representative peritumoral and one cancerous region were selected. Scale bar=50 μm.

Similarly, images of three HCC tissue samples (H2, H3, and H4) from other patients were obtained and analyzed, providing FLIM data from the presentative peri-tumoral and cancerous regions of each sample, as illustrated in **Figure 4** and **Supplementary Figures S1**, **S2**. In **Figure 4**, the results showed significant difference in fluorescence life-time characteristics between the two types of regions. The cancerous parts generally exhibited a higher fluorescence lifetime, consistent with previous findings from sample H1. However, the difference between the peritumoral and cancerous region of each sample varied, which might be due to different cancer stages and individual patient status. This phenomenon might be explained by cellular metabolic changes, microenvironment remodeling, and tumor progression.

**Figure 5:**
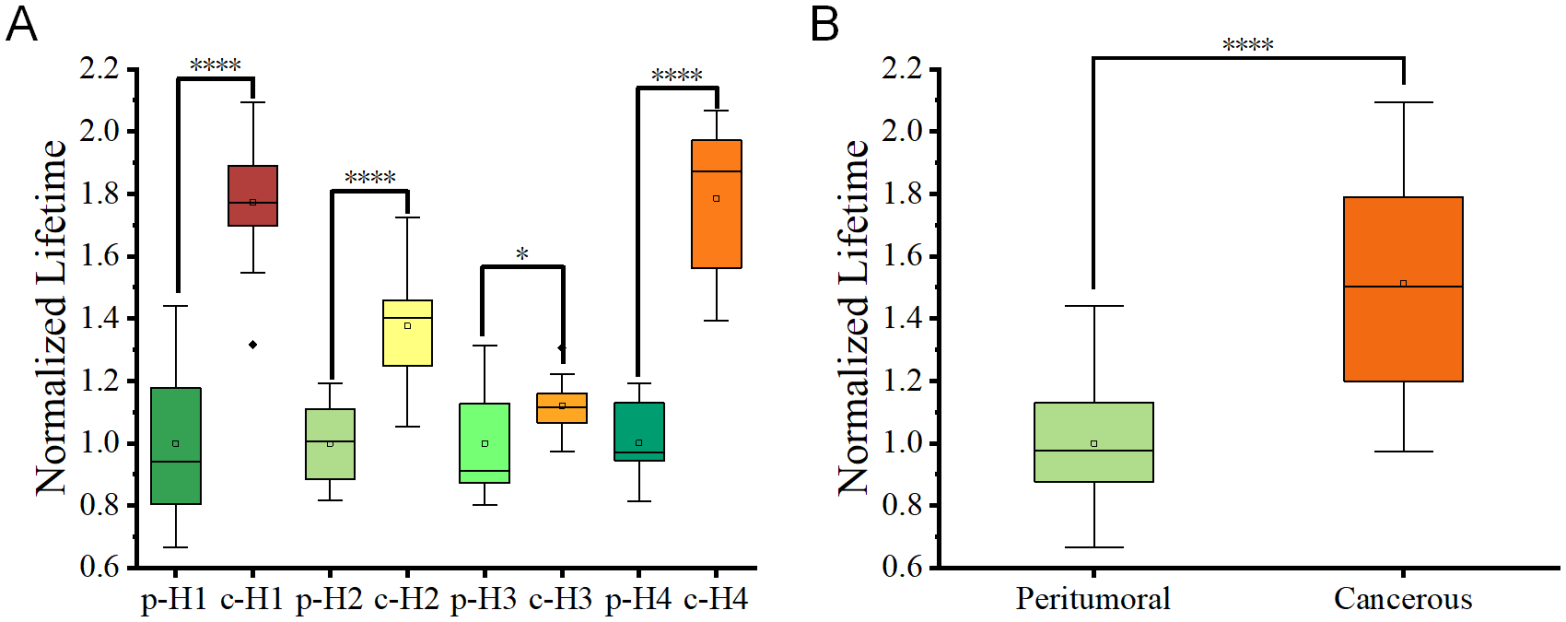
**(A)** Box plots depicting fluorescence lifetime distributions for peritumoral (p) and cancerous c regions in tissue slices from four patients. Each box represented the interquartile range (IQR), and the central line indicates the median value. **(B)** Combined box plots summarizing fluorescence lifetime data for all peritumoral and cancerous regions within the tissue slices. Statistical significance between groups was determined using student t-tests, with p < 0.05 (*) and p < 0.0001 (****).

For better understanding of the experimental results, the averages value of the peritumoral regions of each sample was arbitrarily set as 1. The normalized ratios were generated from the lifetime values of both the peritumoral regions and the cancerous regions divided by these average values. **Figure 5A** showed the normalized lifetime values of the total four sample sections (H1-4). Additionally, **Figure 5B** was a comparison combining all peritumoral and cancerous data, which presented a significant difference in normalized fluorescence lifetime values between peritumoral and cancerous regions from all samples. This comparison indicated that FLIM approach could effectively distinguish the two types of tissue sections.

In addition to providing fluorescence intensity images and fluorescence lifetime distribution histograms, FLIM offers a more accurate processing method called the phasor plot. The phasor plot organizes pixels based on their similar lifetimes. This method uses frequency-domain calculations, which improve accuracy by eliminating the need to fit photon counts directly to curves. One representative fluorescence life-time pseudo-colored image from either the peritumoral region or the cancerous region of sample H1 was selected for segmentation using the phasor plot analysis.

**Figure 6:**
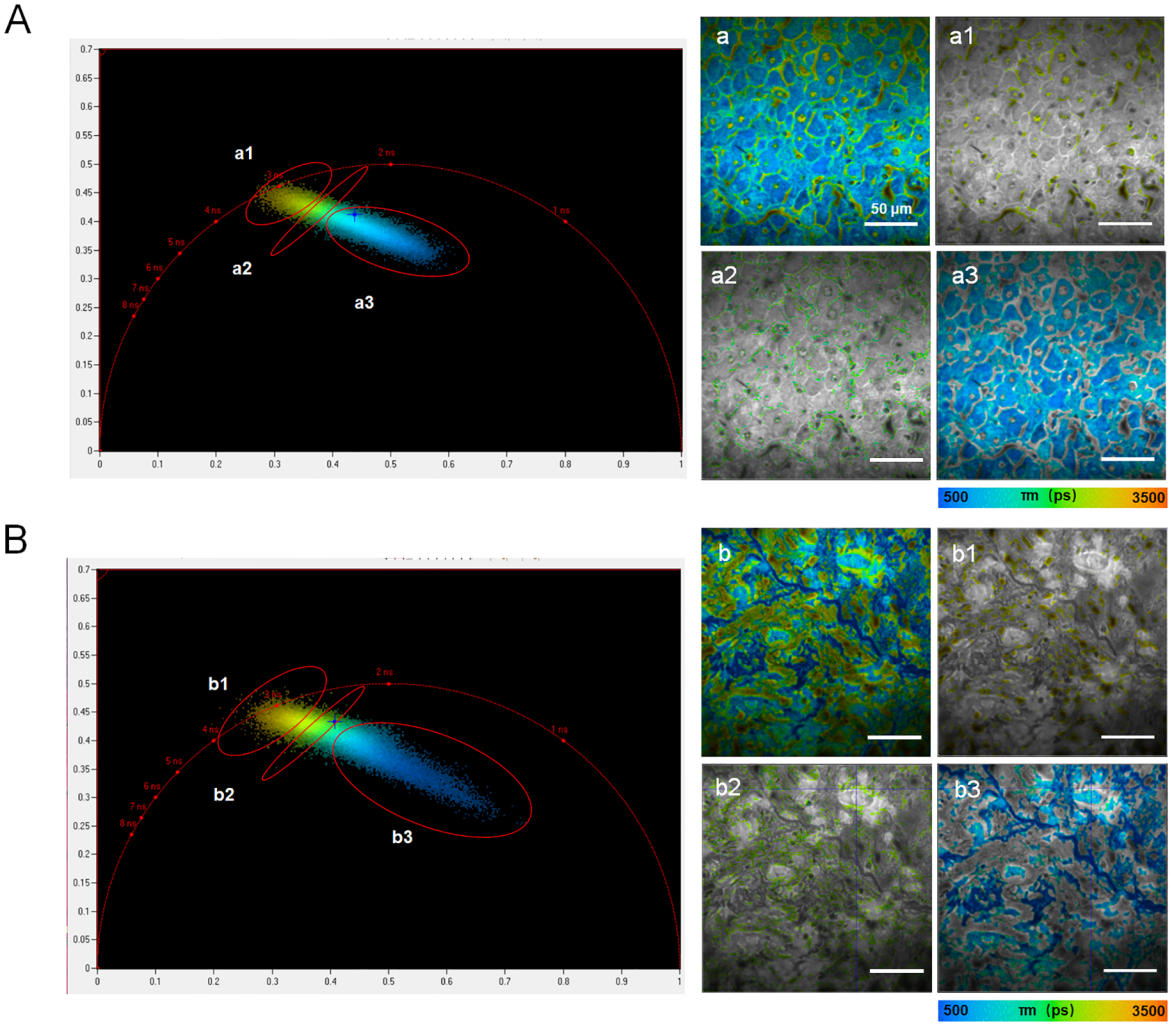
Phasor plots processing in the peritumoral region **(A)** and cancerous region **(B)** from sample H1. The left panels showed phasor plot analysis, with clusters labeled a1–a3 or b1–b3 identified in polar coordinates, representing groups of pixels with similar fluorescence lifetimes. The right panels displayed the corresponding segmentation results for the fluorescence lifetime images shown in the left panels. The scale bar in each image in the right parts equaled to 50 μm.

In terms of cellular structure, the segmentation results analyzed with phasor plot were shown as in **Figure 6**. The fluorescence lifetimes follow a descending order. In the peritumoral region, the average fluorescence lifetimes are 2530.2 ps, 1990.9 ps, and 1040.5 ps for a1, a2, and a3, respectively. In the cancerous region, the average fluorescence lifetimes are 2547.3 ps, 2258.8 ps, and 985.5 ps for b1, b2 and b3, respectively. These results indicated significant variations in cellular composition between cancerous and peritumoral regions.

According to **Table 1**, The lifetime ratio of cancerous to peritumoral regions correlates with bilirubin levels: 0.79 for total bilirubin, 0.87 for direct bilirubin, and 0.75 for indirect bilirubin. This analysis indicated that FLIM data of the ratio of cancerous regions to adjacent peritumoral regions in H&E-stained samples might be related with some important pathological factors. The significant differences in fluorescence lifetime distributions between cancerous and peritumoral regions emphasized their distinct molecular and metabolic characteristics. In conclusion, FLIM method can provide significant potential as a complementary tool to traditional histopathology for the diagnosis of HCC.

In summary, this work has compared liver cancer tissue with peritumoral tissue with FLIM approach. The results demonstrated that the average fluorescence lifetime values of liver cancer tissue were significantly higher than those of peritumoral tissue, indicating statistically difference both median and mean values. Statistically significant differences were observed in both the median and mean values. These data under-scored the unique fluorescence characteristics of liver cancer tissue, confirming the sensitivity and reliability of FLIM in deafferenting cancerous tissue from normal tissue. The study has provided strong evidence for the application of FLIM in the diagnosis of cancer, providing a potential foundation for the development of related optical diagnostic tools.

In future work, incorporating a larger number of clinical cases and utilizing–AI-based analytical methods (29) may help address current limitations and thereby accelerate the clinical translation and applications of FLIM in liver cancer diagnosis.

## Data Availability

The original contributions presented in the study are included in the article/**Supplementary Material**. Further inquiries can be directed to the corresponding authors.
