# Supplementary material for: Fluorescence lifetime imaging microscopy approach reveals quantitative signatures for hepatocellular carcinoma diagnosis
Source: Front Oncol. 2025 Jul 29;15:1598334. doi: 10.3389/fonc.2025.1598334 (PMC12339318; doi:10.3389/fonc.2025.1598334)
Supplement: Supplementary file 1 [file Supplementaryfile1.docx]

Supplementary Material

# Supplementary Figures


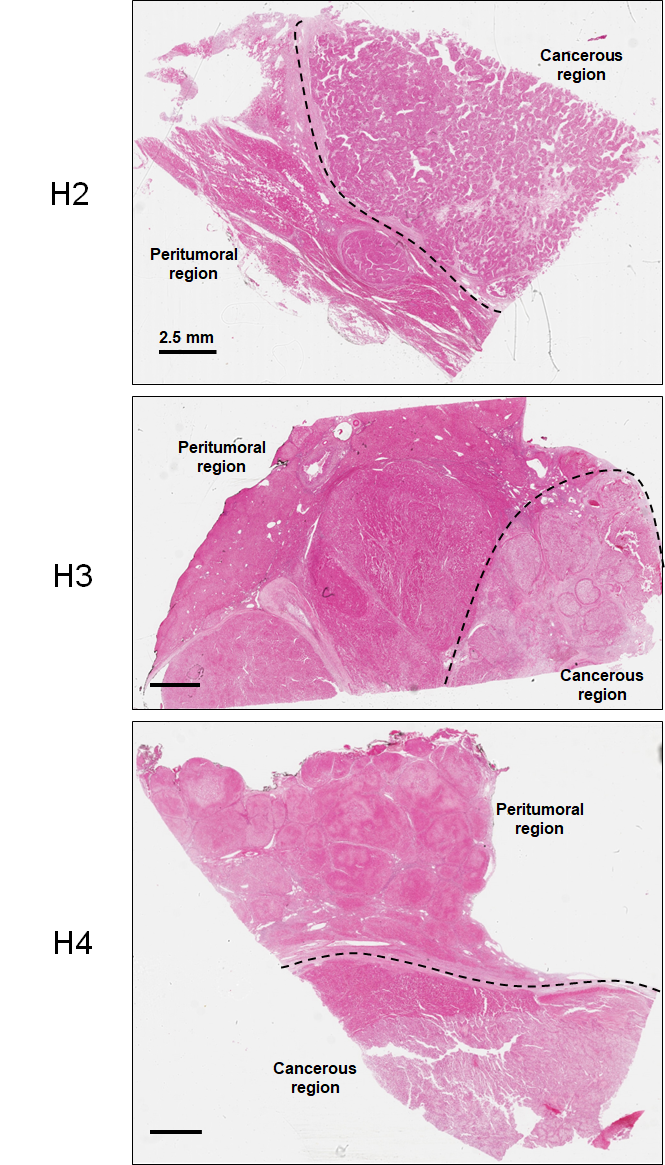


**Supplementary Figure 1.** The whole-slide scanned images of the other three samples (H2, H3, H4). Each section included the cancerous region and the peritumoral region. Scale bar=2.5 mm.


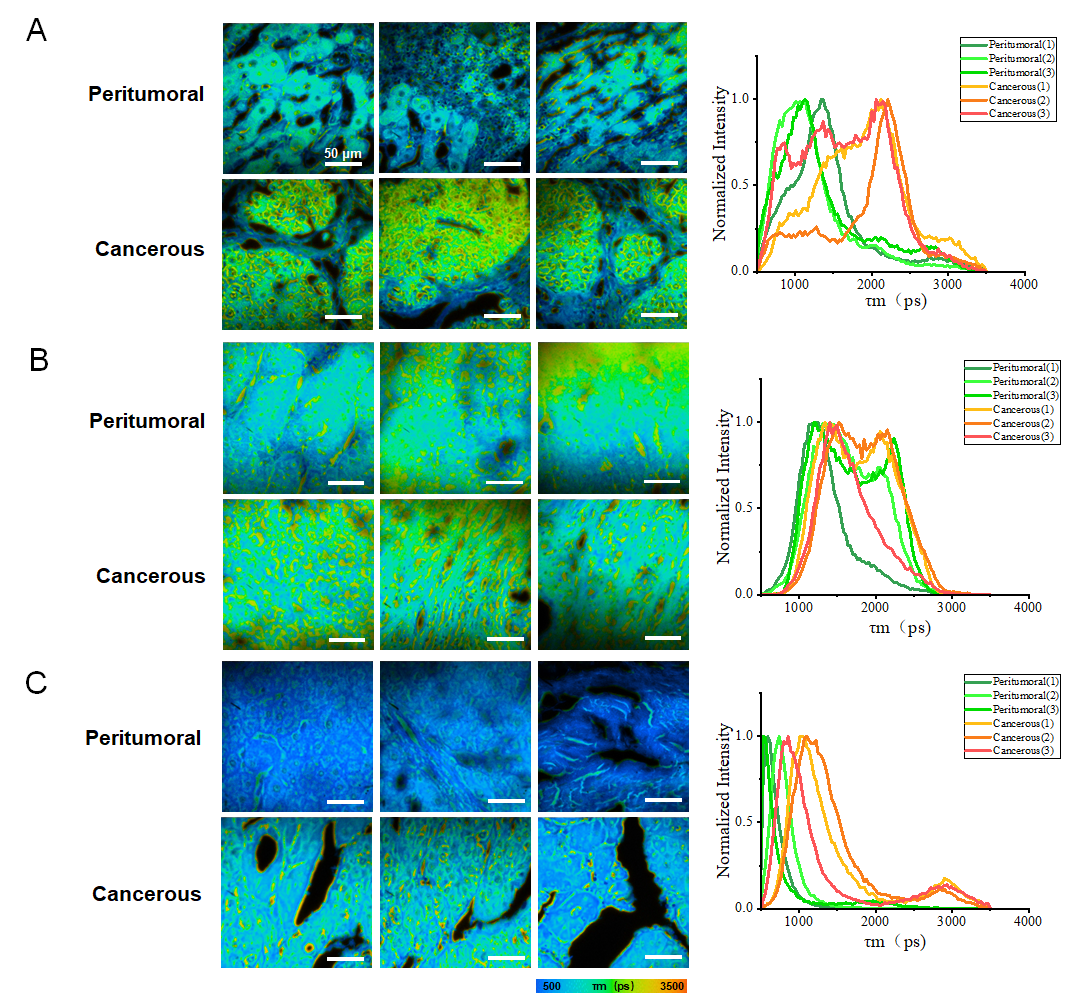


Supplementary Figure 2. FLIM data of hepatocellular carcinoma (HCC) tissue sections. A, B, and C represented samples H2, H3, and H4, respectively. The left column of each row illustrated pseudo-colored FLIM images of both peritumoral and cancerous regions from each sample. The right column of each showed the corresponding fluorescence lifetime distribution histograms of each sample, where distinct curves presented different tissue types. Scale bar=50 μm.


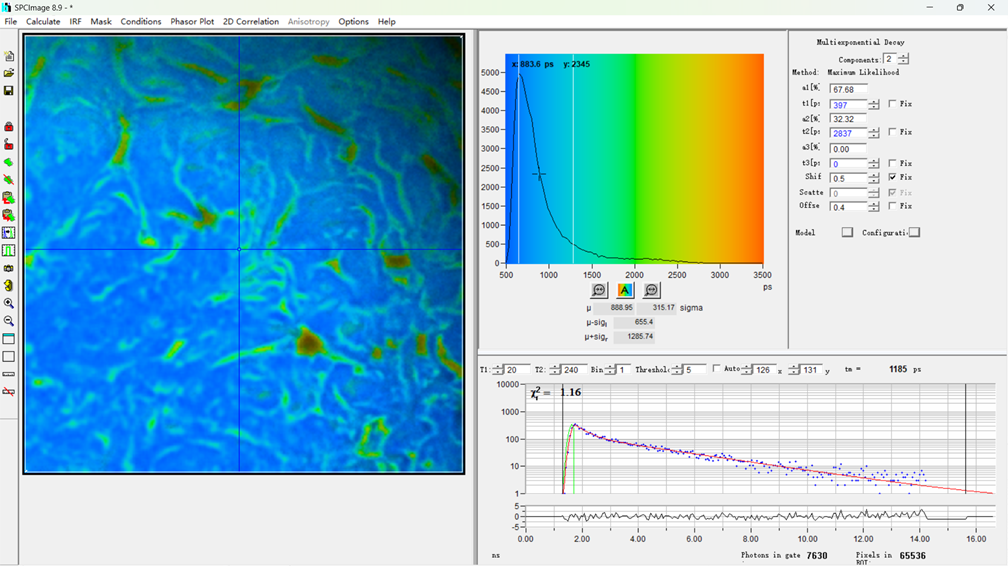


**Supplementary Figure 3.** A typical Analysis interface of the SPCImage software for analyzing FLIM data. The left figure displayed a pseudo-colored FLIM image, where different colors represented different lifetimes: blue indicated shorter lifetime values, while red and green corresponded to longer lifetime values. The upper-middle figure presented the corresponding fluorescence lifetime distribution histogram, showing the distribution of fluorescence lifetimes across different lifetime values. The upper-right panel showed a biexponential decay fitting, yielding two lifetime components (τ1 = 397 ps, τ2 = 2837 ps) and their respective fractions, analyzed by using the Maximum Likelihood Method. The lower-right panel displayed the fluorescence decay curve, where the experimental data (blue) aligned well with the fitted curve (red), with χ² = 1.16, indicating a satisfactory fit.
